# Cardiac fluid dynamics meets deformation imaging

**DOI:** 10.1186/s12947-018-0122-2

**Published:** 2018-02-20

**Authors:** Matteo Dal Ferro, Davide Stolfo, Valerio De Paris, Pierluigi Lesizza, Renata Korcova, Dario Collia, Giovanni Tonti, Gianfranco Sinagra, Gianni Pedrizzetti

**Affiliations:** 1 Cardiovascular Department, Azienda Ospedaliera Universitaria Integrata of Trieste, Trieste, Italy; 2 Department of Engineering and Architecture, University of Trieste, P.le Europa 1, 34127 Trieste, Italy; 3 Cardiology Division, ‘G. D’Annunzio’ University, Chieti, Italy

**Keywords:** Cardiac fluid dynamics, Speckle tracking, Deformation imaging, Hemodynamic forces, Intraventricular pressure gradient

## Abstract

Cardiac function is about creating and sustaining blood in motion. This is achieved through a proper sequence of myocardial deformation whose final goal is that of creating flow. Deformation imaging provided valuable contributions to understanding cardiac mechanics; more recently, several studies evidenced the existence of an intimate relationship between cardiac function and intra-ventricular fluid dynamics. This paper summarizes the recent advances in cardiac flow evaluations, highlighting its relationship with heart wall mechanics assessed through the newest techniques of deformation imaging and finally providing an opinion of the most promising clinical perspectives of this emerging field. It will be shown how fluid dynamics can integrate volumetric and deformation assessments to provide a further level of knowledge of cardiac mechanics.

## Background

The evaluation of blood flow velocity inside the heart has a pivotal role in all echocardiographic studies. For example, the pattern of pulsed wave Doppler (PW) of mitral inflow, when combined with other measurements, allows a good estimate of the diastolic function of left ventricle (LV), moreover Doppler derived pressure gradient (PG) across the valves and Color Doppler representation of flow are currently used to estimate valvular stenosis o regurgitation. Evolving technologies allow now a deeper access to the three-dimensional pattern of blood motion inside the heart and recent literature unveil novel, startling, physiological aspects of cardiac fluid dynamics.

This paper aims to give a thoughtful summary of the latest advances in cardiac flow evaluations. The overall literature in this field is multidisciplinary and a thorough review of the many aspects involved is out of the scope of this manuscript. Here, literature and recent imaging methods are used for driving the reader toward prospective clinical applications of cardiac fluid dynamics, highlighting the relationship with wall mechanics assessed through the newest techniques of deformation imaging. The eventual objective of this paper is that of providing an opinion of the most promising clinical perspectives of this emerging field.

To this aim, the manuscript is organized in an unconventional way. It starts with a review of the main lines of research in cardiac fluid dynamics, deformation imaging and their interrelation. However, fluid dynamics and myocardial deformation are found in literature as separate topics only. Therefore, the paper proceeds presenting preliminary results of two original applications, as instructive examples where both flow and strain are analyzed at the same time. Afterwards, a unitary discussion reporting authors’ viewpoint is provided.

## Lines of research in literature

In a pioneering letter to Nature [1] the asymmetric sinuous flow paths around a vortex in the human left ventricle (LV) were accurately described using magnetic resonance sequences. It was suggested therein that the observed asymmetric vortical arrangement of flow was the functional counterpart of the looped heart structure that enhances the atrium-ventricular mechanical synergy supporting the transfer of momentum from the entry mitral jet to the systolic ejection. The combination of physics and physiology allowed revealing the dynamical balance between LV asymmetrical shape, vortex formation and longitudinal filling-emptying mechanism [2]. Following these initial observations, more recently, several studies evidenced the existence of an intimate relationship between cardiac function and the behavior of intra-ventricular fluid dynamics. Based on these findings most researchers initially paid particular attention to the risk of thrombus formation due to the increased time of blood persistence in the LV associated with wall motion abnormalities from any causes [3–5]. More recently, attention was directed toward the efficiency of blood-tissue dynamical interaction [3, 6] as a way to detect pathological conditions well before overt clinical manifestation, in a phase during which appropriate therapeutic interventions can prevent the progression of the disease or even reverse its outcome [7, 8]. The latter topic will be careful discussed here for its potential clinical relevance that requires thoughtful and knowledgeable developments.

### Initial clinical applications of cardiac fluid dynamics

Blood is an incompressible medium that interacts with the surrounding tissue by the exchanges of forces and momentum in consequence of the displacement of tissue regions. In different terms, tissue deformation creates intraventricular pressure gradients (IVPGs) that drive blood motion and, the other way round, flow-mediated IVPGs create forces on the tissue that affect its deformation. The physiological relevance of IVPGs was recognized in catheterized animal models since long time and their alteration was demonstrated in dysfunctional and failing hearts [9, 10]. Since recently, technical advancements in flow imaging method allow the non-invasive evaluation of flow forces, also termed hemodynamic forces, which are the IVPGs averaged over the LV volume thus opening the possibility to test directly the usefulness of flow force assessments in clinical scenarios.

First echocardiographic flow analyses were done using intravenous microbubbles contrast agents, whose rheology is the same of blood particles, that can be easily detected by ultrasound imaging and then tracked by means of image analysis methods (Echo-PIV) [11]. Particular relevance was gained by recent Echo-PIV studies aimed to verify the association of flow imbalance with the risk of LV remodeling. This research line was grounded on the observations that, under normal conditions, flow forces are aligned along the base-apex direction, in compliance with the emptying-filling cyclic path. This natural dynamic flow alignment is invariably altered in any pathological condition connoted by anomalies of the spatial and/or temporal course of the segmental dynamics of the cardiac walls. For this reason, it was hypothesized that the occurrence of non-physiological transversal components of flow forces can represent an important sign of abnormal cardiac function and even predict the triggering of adaptive mechanisms. To test this conjecture, a population of volumetric responders to cardiac resynchronization therapy (CRT) was studied; they represent a unique physio-pathological model thanks to the possibility of estimating intra-individual variations of the intracavitary PG pattern associated with the interruption/activation of the resynchronizing stimulation [12]. Results demonstrated that flow forces are properly aligned with the LV axis under pacing therapy, while they are altered as soon as pacing is interrupted. Conversely, all patients who do not respond to CRT show preeminent transversal course of PG either during pacing or in basal conditions demonstrating that the response to therapy is always associated with improvement/ /normalization of LV flow dynamics. These observation also support the hypothesis that alteration of a flow alignment can be causally related to LV remodeling [13].

These preliminary studies were soon followed by three-dimensional phase-contrast MRI (often referred as 4D flow MRI), which confirmed that flow forces are consistently aligned with the LV axis in normal subjects while they are noticeably altered in dilated and dysfunctional hearts [14, 15]. 4D Flow MRI also confirmed previous Echo-PIV results that conduction abnormality in heart failure patients with left bundle branch block (LBBB) correlates with deviation of flow forces, and suggest that the latter may be predictive for the response to CRT [16].

Consensus is growing about the relevance of blood motion to the heart physiology and as a potential predictor of LV remodeling after an acute event or a therapeutic procedure [8, 13]. However, advances on clinical applications based on LV fluid dynamics are limited by the complexity or limited accuracy of cardiac flow imaging methods [17–19]. Early results, although promising, still lack of conclusive clinical proofs providing evidence for end-points addressed by fluid dynamics.

### Speckle tracking and deformation imaging

Recent years have rather experienced the advent of speckle tracking (ST) technology and the following development of cardiac deformation imaging. ST echocardiography led to a novel conception about LV function, described not only in terms of volume change but also of pattern of deformation characterized by longitudinal and circumferential shortening [20]. Based on LV strain measurements, novel pathophysiological classification of heart failure were proposed as those associated with predominant longitudinal dysfunction, with transmural dysfunction affecting longitudinal and circumferential strain, and with predominant circumferential dysfunction [21, 22]. The technological characteristics of ST technology suggest that global longitudinal and circumferential strain (GLS, GCS) are the most reproducible and appropriate parameters for clinical applications [23]. In this respect, ST technology was demonstrated to be mature for clinical applications. Although ejection fraction (EF) remains the primary measure for assessing the presence of systolic LV dysfunction, in numerous clinical conditions strain measurements were considered complementary, sometime more effective, in detecting alteration in LV function [24].

### Speckle tracking and flow imaging

Deformation imaging is progressively entering in the clinical arena for classifying the cardiac function with increased accuracy [20, 25]. Differently, flow imaging provides new physiological insights and promises to be able to detect functional alterations before tissues have undergone to evident, sometime irreversible, modifications. Therefore, flow assessments could become complementary to strain, through the promise of a potentially predictive tool of cardiac outcome after acute events or therapeutic procedures.

However, flow and deformation are intimately connected: cardiac function is about creating and sustaining blood motion, which is achieved through a proper sequence of myocardial contraction and relaxation. Thus, flow forms represent a different aspect of tissue motion whose even microscopic changes may alter the distribution of intracardiac flow forces. The two aspects are so intimately linked that from an appropriate knowledge of tissue motion it is possible to estimate the flow forces, or the IVPGs, that develop inside the cardiac chambers [26]. A validation study compared flow forces computed by 4D Flow MRI with the same obtained from a mathematical model that uses endocardial motion on three apical projections and the size of the aortic and mitral orifice demonstrating the accuracy of the model. Therefore, the knowledge of the LV endocardial motion from ST echocardiography, as it is commonly used to evaluate myocardial strain, also allow estimations of flow forces [27].

### Perspectives

Flow imaging technology is primarily represented by 4D Flow MRI. Echocardiography, either Echo-PIV or solutions based on Color-Doppler, is a second option at a lower level in terms of accuracy and reliability [19]; however, novel technological ultrasound solutions could be on their way [28]. Flow imaging methods allow exploring the potential of quantifications based on fluid dynamics.

Flow forces appear currently a promising quantity relating blood motion to cardiac function; it has a rigorous physical significance and appears clinical relevant. Flow forces can be estimated by ST thus their extensive clinical validation becomes relatively easy.

**Fig. 1:**
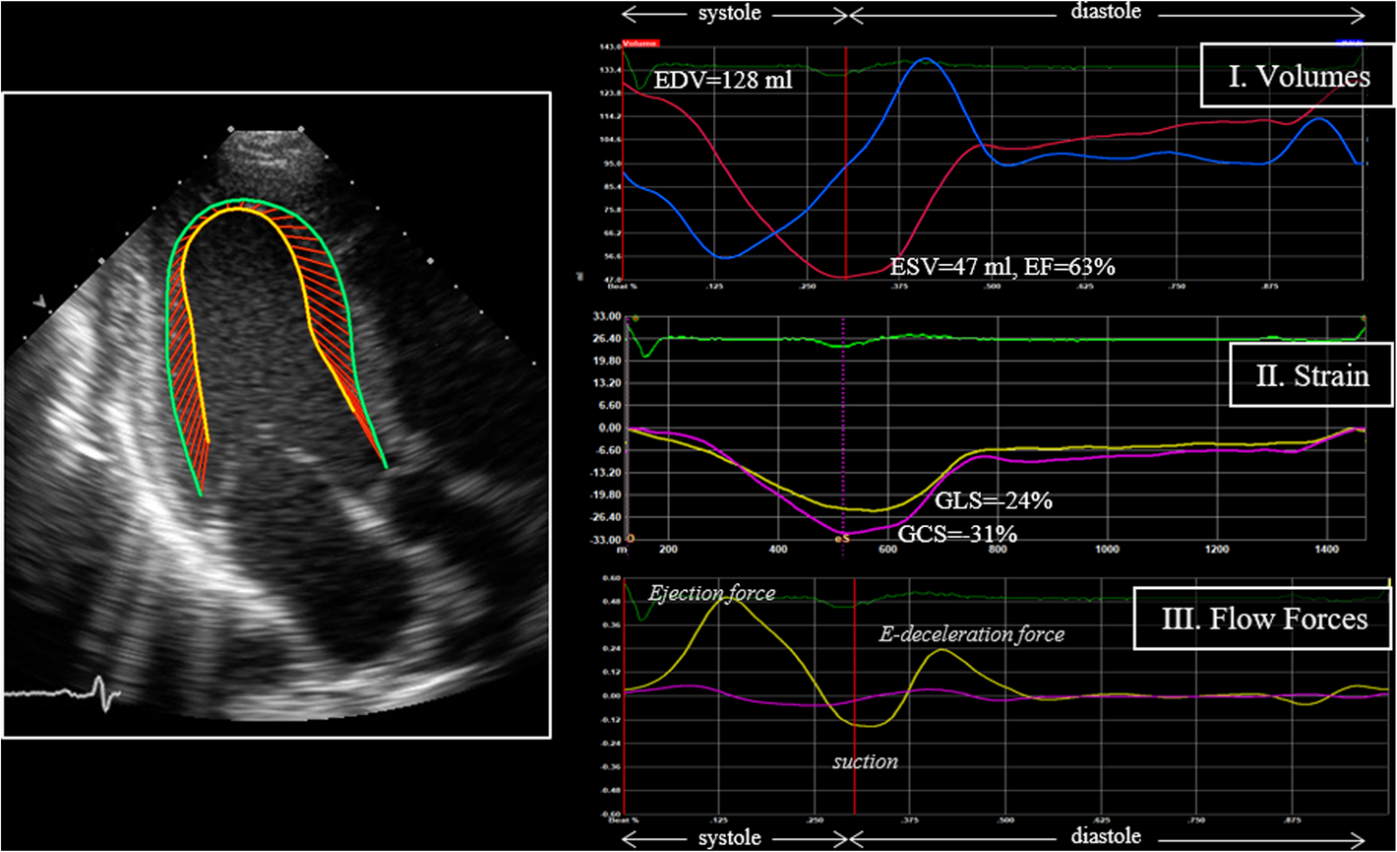
The knowledge of endocardial borders in regular B-mode (left side, is an generic exemplary image) along the entire heartbeat can be used for a comprehensive assessment of cardiac mechanics based on volumes, deformation, and flow (right side). Volume curve and EF provide a primary measure of cardiac function; strain represents a second level of information whose clinical value was demonstrated in literature; a flow force provides a further level of knowledge

The knowledge of endocardial borders has a long history for the evaluations of volumetric measures and EF. Since the advent of ST technology, the same endocardial borders can be used to evaluate strain. Volumes and EF provide a primary measure of cardiac function; strains represent a second level of information integrative to volumetric measures. Evaluation of flow forces from ST provides a further level of knowledge that is incremental to volumes and strain. These three levels of information are shown in Fig. 1 for a normal subject.

The time profiles of strain curves are somehow comparable to the volume curve and it was natural to use end-systolic strain values as clinical parameters. The time profile of the flow forces brings largely new information and the definition of most appropriate clinical parameters in the different clinical situations is still under development. Two applications are reported below as illustrative examples of the 3-level evaluations based on volumes, strain, and flow forces.

## Exemplary clinical cases

The previous literature review eventually highlighted that blood flow and tissue deformation represent two faces of cardiac function and therefore they are deeply interrelated. However, flow and strain are found in literature as separated topics only. We introduce here preliminary results of an integrated analysis where both aspects are evaluated at the same time. This approach is presented in two clinical populations: patients with cardiomyopathy and few subjects who underwent cardiac resynchronization therapy (CRT), with the objective of showing the application of the above concepts into real clinical scenarios. Discussing the clinical relevance of the finding is beyond the scope of this manuscript; these analyses are shown by way of example with the aim of displaying means of integrating measurement based on flow and strain for reaching a deeper understanding of cardiac mechanics at the individual clinical level.

### Methods

First, we retrospectively analyzed a population of 33 subjects composed of 13 healthy volunteers (Controls), 9 patients with dilated cardiomyopathy (DCM), and 11 patients with obstructive hypertrophic cardiomyopathy (OHCM), consecutively enrolled in Trieste Heart Muscle Disease Registry [29, 30] between 2008 and 2015. Briefly, diagnosis of DCM and OHCM was defined according to current criteria [31, 32] and all patients underwent extensive clinical and laboratory characterization. Patients underwent a complete echocardiographic evaluation at baseline and periodically during follow up. The average volumetric and strain parameters are reported in Table 1.

**Table 1:** Mean volumetric and deformation parameters of first population

	Controls	DCM	OHCM
EDV [ml]	105 ± 24	233 ± 69	131 ± 47
ESV [ml]	45 ± 12	179 ± 48	44 ± 16
EF [%]	57 ± 6	22 ± 8	65 ± 5
GLS [%]	−20 ± 3	−7 ± 2	−18 ± 3
GCS [%]	−27 ± 5	−8 ± 4	−34 ± 5

For a second test, 3 patients fulfilling criteria of responders or super-responders to cardiac resynchronization therapy (CRT) [13], were randomly extracted from the CRT registry of our Institution and compared to 3 patients non-responders to CRT [33]. Patients underwent a complete echocardiographic study before implantation (PRE) and few months after the procedure (POST). Table 2 reports the volumetric measures. All patients were then followed-up for a mean period of 3 ± 1 year to assess the long-term effects of resynchronization.

**Table 2:** Volumetric parameters of CRT population

		EDV [ml]			ESV [ml]			EF [%]	
		PRE	POST	Δ%	PRE	POST	Δ%	PRE	POST
1	Resp	210	108	−48%	175	70	−60%	17%	35%
2	Resp	146	69	−53%	103	38	−63%	29%	45%
3	Resp	263	201	−23%	174	98	−43%	34%	51%
4	NR	125	152	+22%	96	113	+18%	23%	26%
5	NR	230	219	−5%	194	185	−5%	16%	16%
6	NR	162	124	−23%	128	87	−32%	21%	30%

The entire study was performed in accordance with the Helsinki declaration; all subjects provided written informed consent (N.O 43/2009, prot 2161).

During the echocardiographic evaluations, the three echocardiographic apical long axis views (4-ch, 3-ch, and 2-ch) were recorded for offline analysis. Image analysis was performed by a commercially available tool (2D-CPA v.1.3; TomTec Imaging Systems Gmbh, Unterschleissheim, Germany). This ST tool requires drawing the end-systolic (ES) endocardial borders and estimates the border over the entire heartbeat; it then allows correcting the end-diastolic (ED) one and propagates the correction accordingly over the entire cycle without affecting the previously drawn ES border. Therefore, it gives full control of ES and ED borders from which the EF and GLS are computed as by guidelines [34]. From the same endocardial borders, the LV diameters from base to apex are evaluated and their reduction from ED to ES, averaged of the LV length, gives the GCS. The apical approach to GCS could be less accurate because the entire circumference is not visible from the apical views; this criticality is minimized by using a triplane evaluation thus applying the same approach and the same approximation commonly used in the evaluation of LV volumes. This approach to circumferential strain is more similar to that used in 3D echocardiography because the border follows the tissue during its longitudinal motion and reduce artifacts in deformation such as those that may result from through-plane displacements of 3D geometry that sometime affect the short axis transversal projections [23, 35]. All evaluations are performed combining the three apical views. Through this approach the EF and the two global strain, GLS and GCS, are evaluated in a consistent way from the same endocardial border calculations.

Longitudinal and radial displacements, which are described by GLS and GCS, respectively, jointly contribute to the volumetric reduction and to ejection fraction (EF). A relationship to estimate the value of EF from those of myocardial strain was presented in [36] where a further dependence on the average LV diameters and thickness was present. That approach can be recast in simpler terms for endocardial strain values proving the explicit relationship

1 $$ \mathrm{EF}=1-\left(\mathrm{GLS}+1\right){\left(\mathrm{GCS}+1\right)}^2. $$

This relationship will be used in the analysis of results for demonstrating how longitudinal and circumferential functions combine to volumetric reduction in the different clinical conditions.

The same ST data are then used to evaluate the hemodynamic forces associated with blood flow. A previous study [27] demonstrated that flow forces (which is a synonymous for “hemodynamic forces”, “flow momentum” or “average IVPGs” also used in literature) can be estimated from the knowledge of the LV geometry and endocardial velocities, obtained by ST, plus the area of the aortic and mitral orifices. The complete mathematical details of the method for transforming endocardial dynamics into flow forces are reported elsewhere [27] and the concept is only quickly summarized here. The total hemodynamic force, ***F***(*t*), exchanged between blood and tissues can be computed by the balance of momentum inside the volume *V*(*t*) of the LV

2 $$ \boldsymbol{F}(t)=\rho {\int}_{V(t)}\frac{\partial \boldsymbol{v}}{\partial t} dV+\rho {\int}_{S(t)}\boldsymbol{v}{v}_n dS, $$

where *S*(*t*) is the surface bounding the volume and ***v*** is the velocity vector where the subscript _*n*_ indicates the outward normal component. The second term in the right-hand-side of the previous formula represents the flux of momentum across the instantaneous LV volume boundary. It can be computed from the velocity at the LV endocardium and at the base, which are known from ST, plus the mean velocity across the mitral and aortic valve, during diastole and systole, respectively, which are the volume rate divided by the valve area. The first term is blood inertia and can be rewritten through the rate of change of the average velocity inside the LV. The longitudinal component of the average velocity can be estimated from mass conservation given the variation of LV shape, known from ST. The main transversal (inferolateral-anteroseptal) components is some more complicated but can also be estimated from ST data accounting for the transit from inflow to outflow; the other transversal component is mainly due to the orientations of the mitral jet and is not considered here. This approach will be used in the analysis of result to integrate to the volumetric and deformation information with those related to cardiac fluid dynamics.

### Results

**Fig. 2:**
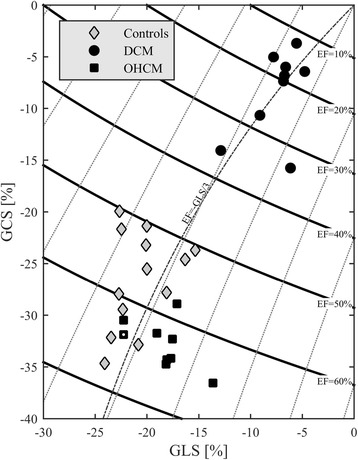
Strain properties in the first population. Curved bands in the GLS-GCS plane represent the regions with constant value of EF. DCM patients present a reduction of EF and of both strain parameters with respect to the Controls. OHCM patients present a conserved or slightly increased EF with respect to controls, thus they tend to displace on the right along the curves of constant EF, which correspond to a reduction of GLS and slight increase of GCS. The OHCM patient indicated with a white dot corresponds to that with highest obstruction

Strain properties for the first population (Control, DCM, OHCM) are summarized in Fig. 2 in the plane GLS-GCS. From the relationship of formula (1), the curves at constant EF can be drawn on this plane showing regions with same EF value obtained with a different combination of longitudinal and circumferential strain. As expected, the DCM patient, that have reduced EF, also present a reduction of contraction in both strain and are displaced toward the origin with respect to Controls. OHCM patients, that have preserved EF, present a tendency to displace toward the right along the curves with constant EF; this corresponds to a reduction of GLS accompanied by a small increase of GCS to ensure preservation of EF.

**Fig. 3:**
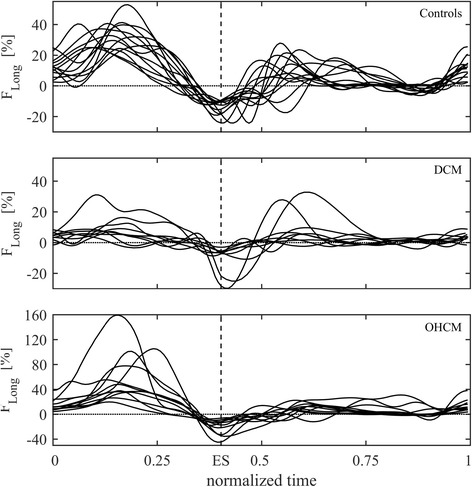
Longitudinal flow forces profiles in the first population. DCM patients present a reduction of flow forces (already normalized with volume). OHCM patients present flow forces with comparable entity than Controls, although with a larger variability (notice the scale difference in the third graph)

**Fig. 4:**
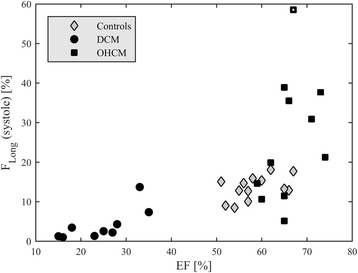
Systolic ejection force (or normalized force impulse) in the first population. DCM patients present a reduction of flow force. Most OHCM patients present OHCM patients present flow forces with comparable entity than Controls with the exceptions of few cases. The OHCM patient indicated with a white dot corresponds to that with highest obstruction

The complete time profiles of the longitudinal (base-apex) component of the flow forces in dimensionless form (normalized with volume and expressed in percentage of gravity acceleration or, equivalently, of the weight of the LV blood volume) are reported in Fig. 3 for the three groups. Time scale is adjusted individually, with a common heartbeat frequency and a common ES instant, just improve visual comparability in the graphic representation. All Controls present a consistent pattern of flow forces, DCM patients confirm a depressed function in terms of flow forces as well, OHCM patients present a significant variability where most patients have approximately normal amplitude and a few patients display higher systolic fluctuations. Transversal forces (not shown here) are comparable in the three groups. A synthesis of the overall fluid dynamics differences is given in Fig. 4 where the mean systolic force (or flow impulse [37]) is reported versus EF. In this set, the higher impulse in OHCM patients correspond to higher septal thickness and higher pressure gradients in the outflow tract. The patient with highest impulse, four times higher than in Controls, was reviewed retrospectively to check whether this could correspond to a clinical peculiarity. This patient indeed presented the highest septal thickness (40 mm) in the OHCM group, was asymptomatic, and the only one carrying a mutation of the gene MYH7.

**Fig. 5:**
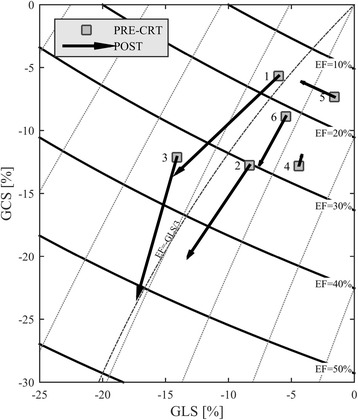
Strain properties in the CRT patients. Curved bands in the GLS-GCS plane represent the regions with constant value of EF. Responder patients (1–3) present an evident improvement in EF and strain values increases accordingly. Non-responder patients (4–6) display minor improvement in EF and strain

**Fig. 6:**
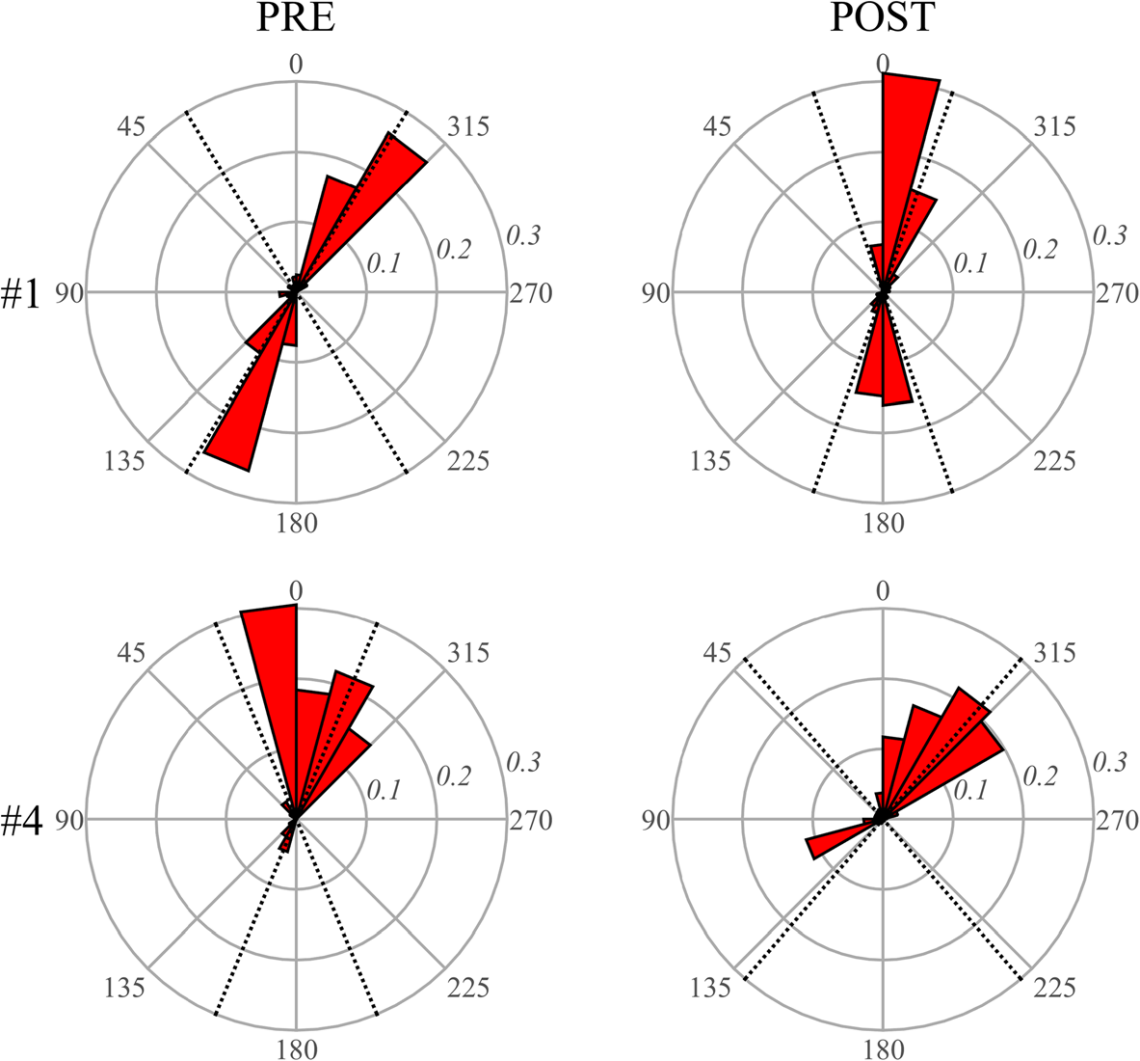
Polar histogram of the distribution of flow forces during systole. Results are reported PRE- and POST-CRT for a responder patient (#1) and a non-responder (#4), the transversal scale is magnified (2×) to improve visual readability. This representation displays that the therapeutic improvement in alignment of flow force is found in the responder patient only

**Fig. 7:**
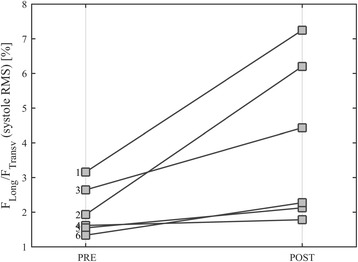
Changes from PRE- and POST-CRT or the ratio between systolic flow force components during systole, measure by root mean square (RMS). Responder patients (1–3) present an evident improvement of force alignment with therapy as indicated by the increase longitudinal-to-transversal ratio. Non-responder patients (4–6) display no or minor improvement in force alignment

The CRT population was analyzed similarly. Figure 5 shows the data in the same GLS-GCS plane, improvement in EF and strain is found in patients who better responded to therapy (1–3). Non-responders (4–6) present different individual behavior with no or limited improvements. Flow forces clearly witness the differences in clinical outcome. Figure 6 shows the polar distribution of flow forces in two patients: the patient who responded to therapy (#1) presents a clear improvement in the alignment of flow forces; the non-responder patient (#4) exhibits a worsening of such a dynamic alignment, which could support the observed slight reduction of strain. Figure 7 summarizes the individual therapeutic outcome in terms of flow forces, confirming and integrating the observations in terms of volumes and strain.

## Discussion

Fluid dynamics provide an alternative viewpoint when looking at cardiac mechanics. Flow forces, or IVPGs, correspond to the ultimate result of LV contraction-relaxation rhythm and, like deformations, play a central role in the description of cardiac function.

The analysis of literature demonstrated a common awareness of the potential relevance of fluid dynamics for clinical assessments; nevertheless, clinical application remained limited. Non-invasive measurements of IVPGs were previously proposed in echocardiography by post-processing of M-mode color Doppler [38–41] or by Echo-PIV [12, 13]; these method present several technical limitations and, also for the unavailability of widespread quantification tools, could not undergo to extensive clinical evaluations. Recently, research in 4D Flow MRI was applied to measuring blood velocities and hemodynamic forces in the heart [14, 15]. Overall, the non-invasive evaluation of hemodynamic force (or IVPGs) appears the most promising clinical application of cardiac fluid dynamics in the short time.

Here we applied a new, previously validated technology that allows quantification of hemodynamic forces using the same ST information used for deformation imaging. This approach, within ST own limitations [23], can highly support diffusion of hemodynamic force measurements by echocardiography. The preliminary clinical applications shown here just by way of example brought evidence that flow force measurements corroborate the findings in terms of volumetric changes and deformations and bring novel incremental information.

Deformation imaging and strain measurements provided an additional level of knowledge of cardiac mechanics with respect to previous evaluations based on volumetric measurement only and parameters like GLS are progressively and firmly entering in the daily clinical practice. Similarly, flow force quantifications is a new field of research promising a further level of knowledge to gain a deeper understanding of cardiac function.

The importance of fluid dynamics, however, may in perspective move beyond the description of cardiac function and extend to the prediction of cardiac outcome. Initial literature results suggest the existence of the intimate relationship between the quality of intraventricular fluid dynamics and longer term geometrical adaptation of the myocardial structure. It was previously suggested [8] that, in LV, endothelial cells are able to sense the loading conditions via shear changes (mechano-sensing), transforming any abnormal condition into adaptive responses (mechano-transduction) [42, 43]. This relationship was previously demonstrated during morphogenesis in embryonic hearts [44, 45]. It was demonstrated that myocardial stretch rapidly activates a plethora of intracellular signaling pathways which decrease the initial load [46]. While extremely efficient, in short time, as a physiological adaption mechanism, under prolonged overstimulation, this process becomes maladaptive, leading to the development of left ventricular hypertrophy and ultimately to heart failure [47]. In this context, flow forces can provide informative content when creating predictive models that can forecast progression or reversal of LV remodeling following therapeutic interventions.

## Conclusion

Fluid dynamics is a promising field of clinical research that can be integrated to volumetric and deformation assessments to provide a further level of knowledge of cardiac mechanics and, possibly, indications of therapeutic outcome. Flow imaging methods like 4D Flow MRI can help to advance research in the field, while novel methods based on ST technology permit an easier access for widespread clinical applications.
